# Responses of Multipotent Retinal Stem Cells to IL-1*β*, IL-18, or IL-17

**DOI:** 10.1155/2015/369312

**Published:** 2015-10-04

**Authors:** Shida Chen, Defen Shen, Nicholas A. Popp, Alexander J. Ogilvy, Jingsheng Tuo, Mones Abu-Asab, Ting Xie, Chi-Chao Chan

**Affiliations:** ^1^Laboratory of Immunology, National Eye Institute, National Institutes of Health, Bethesda, MD 20892, USA; ^2^Zhongshan Ophthalmic Center, Sun Yat-sen University, Guangzhou 510060, China; ^3^Histology Core, National Eye Institute, National Institutes of Health, Bethesda, MD 20892, USA; ^4^Stowers Institute for Medical Research, Kansas City, MO 64110, USA; ^5^Department of Anatomy and Cell Biology, University of Kansas School of Medicine, Kansas City, KS 66160, USA

## Abstract

*Purpose*. To investigate how multipotent retinal stem cells (RSCs) isolated from mice respond to the proinflammatory signaling molecules, IL-1*β*, IL-18, and IL-17A. *Materials and Methods*. RSCs were cultured in a specific culture medium and were treated with these cytokines. Cell viability was detected by MTT assay; ultrastructure was evaluated by transmission electron microscopy; expression of IL-17rc and proapoptotic proteins was detected by immunocytochemistry and expression of *Il-6* and *Il-17a* was detected by quantitative RT-PCR. As a comparison, primary mouse retinal pigment epithelium (RPE) cells were also treated with IL-1*β*, IL-18, or IL-17A and analyzed for the expression of *Il-6* and *Il-17rc*. *Results*. Treatment with IL-1*β*, IL-18, or IL-17A decreased RSC viability in a dose-dependent fashion and led to damage in cellular ultrastructure including pyroptotic and/or necroptotic cells. IL-1*β* and IL-18 could induce proapoptotic protein expression. All treatments induced significantly higher expression of *Il-6* and *Il-17rc* in both cells. However, neither IL-1*β* nor IL-18 could induce *Il-17a* expression in RSCs. *Conclusions*. IL-1*β*, IL-18, and IL-17A induce retinal cell death via pyroptosis/necroptosis and apoptosis. They also provoke proinflammatory responses in RSCs. Though IL-1*β* and IL-18 could not induce *Il-17a* expression in RSCs, they both increase *Il-17rc* expression, which may mediate the effect of *Il-17a*.

## 1. Introduction

Age-related macular degeneration (AMD) is a progressive disease characterized by the degeneration of retinal pigment epithelium (RPE) and photoreceptor atrophy in the macula [1, 2]. Inflammation, particularly innate immunity, is implicated in AMD pathogenesis [3]. Recently, the inflammasome, a multimeric protein consisting of nod-like receptor (NLR), apoptosis-associated speck-like domain contains a caspase-recruitment domain (ASC), and pro-caspase-1 plays a central role in innate immunity and has been implicated in the pathogenesis of AMD [4, 5]. Activation of the NLRP3 inflammasome results in caspase-1 cleaving pro-IL-1*β* and pro-IL-18 into their mature proinflammatory forms in macrophages and RPE cells [5, 6]. However, the direct effect of IL-1*β* and IL-18 on other retinal cells has not been well studied.

In combination with IL-23, IL-1*β* or IL-18 can induce interleukin-17A (IL-17A) production by Th17 cells, *γδ* T cells, and iNKT cells [7–10]. Growing evidence has implicated IL-17A involvement in AMD pathogenesis. Higher levels of IL-17A are found in the serum and macular tissues of the AMD patients when compared to age-matched controls [11, 12].* In vitro*, IL-17A is cytotoxic to ARPE-19 cells, characterized by the accumulation of cytoplasmic lipids, autophagosome formation, and the presence of cleaved caspase-9 and cleaved caspase-3 [12]. IL-17RC, a member of IL-17R family and the primary receptor for IL-17A, is highly expressed in AMD macular tissues and in ARPE-19 cells [12]. In a study of twins with discordant AMD status, hypomethylation of the IL-17RC promoter was found in those with AMD. This finding was correlated with elevated expression of IL-17RC in peripheral blood cells as well as the macular tissue of AMD patients [13]. However, the direct effect of IL-17A on other cell types remains to be explored.

To test the hypothesis that IL-18 and IL-1*β* could stimulate IL-17A secretion in retinal cells, we used a mouse-derived multipotent retinal stem cell line (RSCs) as a model. RSCs are cultured stem cells from the mouse retina and can be efficiently differentiated into photoreceptor cells and all major cell types of neural retina under optimized differentiation conditions [14]. Subretinal injection of these differentiated photoreceptors into slowly degenerating* rd7* mouse eyes can form new synapses with resident retinal neurons; in fast degenerating* rd1* mouse eyes, injection of these cells can restore light response. These findings suggest that human retinal or neuronal stem cells could be useful for treating retinal degeneration in AMD [14]. We stimulated RSCs with IL-1*β*, IL-18, or IL-17A and characterized the inflammatory and cytotoxic responses.

## 2. Materials and Methods 

### 2.1. Cell Culture and Stimulation

The RSC line was obtained from primary culture of adult CD-1 mouse neuroretina and cultured as described previously [14]. Briefly, RSCs were cultured in medium for retinal stem cells (RCM) composed of DMEM/F12 (1 : 1, Sigma, St Louis, MO, USA), insulin-transferrin-selenium-A supplement (Invitrogen, Eugene, OR, USA), 1.0 g/L bovine serum albumin (BSA, Sigma), 1.0 g/L glucose (Sigma), 1.0 g/L lactose (Sigma), 0.045 g/L proline (Sigma), 11.25 *μ*g/mL linoleic acid (Sigma), 5 mM glutamine (Invitrogen), 2 mM nicotinamide (Sigma), 5% knockout serum replacement (Life Technologies, NY, USA), 20 ng/mL epidermal growth factor (EGF, Millipore, Billerica, MA, USA), and 20 ng/mL basic fibroblast growth factor (bFGF, R&D Systems, Minneapolis, MN, USA). Cells were passaged at 90% confluence using Accutase (Sigma). RSCs grown to 70%–80% confluence were treated with 1–100 ng/mL recombinant mouse IL-1*β* (R&D Systems), recombinant mouse IL-18 (MBL, Woods Hole, MA, USA), or recombinant mouse IL-17 (R&D Systems) for 24 hours.

### 2.2. Culture of Primary RPE Cells

All procedures using animals adhered to the Association for Research in Vision and Ophthalmology statement for the use of animals and the NEI's Institutional Animal Care and Use Committee approved protocols. Mouse RPE was isolated from retinas of C57/B6J mice at 6–8 weeks of age as described previously [15]. Briefly, mice were euthanized, and their eyes were enucleated. The globes were washed with PBS containing 1% penicillin-streptomycin (Sigma) and then were dissected free of periocular connective tissue. Then, the globe was placed on 2% Dispase II (neutral protease, grade II, Roche, Indianapolis, IN, USA) and incubated at 37°C for 40 min. The globe was transferred to DMEM/F12 media, the anterior segment was removed, and the retina containing the RPE layer was dissected free. The loosely adherent RPE cell layer was gently separated from the retina and transferred to a 15 mL tube containing DMEM/F12, 20% FBS, and 1% L-glutamine-penicillin-streptomycin. Cells were then centrifuged at 1000 rpm for 5 min and resuspended. The RPE suspension was added to 6-well cell culture plates. The medium was changed after 5-6 days and every 2-3 days thereafter. The RPE cells between two and three passages were stimulated with 100 ng/mL recombinant mouse IL-1*β* (R&D Systems), 10 ng/mL recombinant mouse IL-18 (MBL), or 10 ng/mL recombinant mouse IL-17 (R&D Systems) for 24 hours.

### 2.3. MTT Assay

The assessment of cell viability was performed using a 3-(4,5-dimethylthiazol-2-yl)-2,5-diphenyl tetrazolium bromide (MTT) assay in RSCs as described previously [15]. Briefly, cells were seeded at 80% confluence to 96-well culture plates. After stimulation with IL-1*β*, IL-18, or IL-17A for 24 hours, cells were washed with PBS and incubated with 20 *μ*L of 5 mg/mL MTT solution (Sigma) for 4 h at 37°C. The medium was aspirated and 200 *μ*L DMSO was added to each well. Plates were then shaken for 15 min at room temperature. Cell viability was determined by measuring the optical density at 570 nm using an ELISA plate reader (BioTek, Burlington, VT, USA). Cell viability represented the optical density ratio of stimulated cells relative to that of unstimulated cells.

### 2.4. Transmission Electron Microscopy

For transmission electron microscopy (TEM), cells were fixed in glutaraldehyde (2.5%, PBS buffered) for 24 hours, then suspended in warm low-melting point agarose (1.5%), pelleted down, and refrigerated overnight at 4°C; solidified pellets were rinsed with PBS three times, doubly-fixed with osmium tetroxide, rinsed again three times with PBS, dehydrated in ethanol, and embedded in Spurr's epoxy resin. Ultrathin sections (100 nm) were mounted on 200 lines/inch copper grids, double-stained with uranyl acetate and lead citrate, and viewed with a JEOL JEM-1010 transmission electron microscope.

### 2.5. Immunocytochemistry

The cells were seeded into 2-well chamber slides, and stimulation was performed at 70% confluence. After stimulation, cells were fixed with acetone, blocked with 1% BSA, and incubated overnight with the following primary antibodies: rabbit anti-mouse FasL (1 : 100, Santa Cruz, Dallas, Texas, USA); rabbit anti-mouse Fas (1 : 100, Santa Cruz); rabbit anti-mouse cleaved caspase-3 (1 : 200, Cell Signaling Technology, Danvers, MA, USA); rabbit anti-mouse cleaved caspase-9 (1 : 200 Cell Signaling Technology). After washing with PBS, secondary antibodies conjugated to either Alexa-488 or Alexa-555 (1 : 500, Invitrogen) were added and incubated for 1 h. After rinsing with PBS, cells were counterstained with 40, 6-diamidino-2-phenylindole dihydrochloride (DAPI, 1 : 1000, Invitrogen) for 5 min. The stained cells were examined under Zeiss 700 Confocal microscope with Zen software.

### 2.6. RNA Isolation and Quantitative RT-PCR

Total RNA was extracted from RSCs by using an RNeasy Mini Kit (Qiagen, Hilden, Germany), and equal amounts of RNA were synthesized to cDNA with Superscript II RNase H Reverse Transcriptase (Invitrogen) according to the manufacturer's instructions. Quantitative RT-PCR (qRT-PCR) was performed using RT² SYBR Green ROX qPCR Mastermix (Qiagen). cDNA was amplified with primers *β-actin*,* Il-6*,* Il-17rc*, or* Il-17a* (Qiagen) separately for 50 cycles. All data were normalized to the *β-actin* mRNA level. Expression fold-changes were calculated by 2^−ΔΔCT^.

### 2.7. Statistical Analysis

Statistical analyses were performed using SPSS version 17.0 (SPSS, Chicago, IL, USA). Unpaired *t*-tests or analysis of variance (ANOVA) were used to compare the difference among different groups. GraphPad Prism 6 software was used to make the figures. A *p* value <0.05 was considered statistically significant.

## 3. Results

### 3.1. Stimulation of the Expression of IL-17RC in RSCs

**Figure 1:**
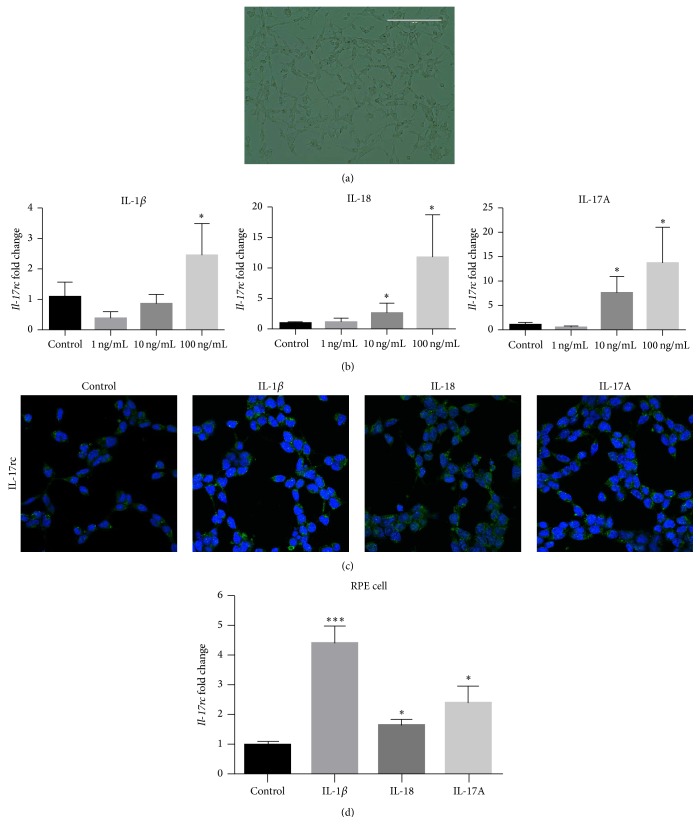
Morphology of the RSCs and Il-17rc expression. (a) RSCs are spindle-shaped even after passaging (scale bar: 200 *μ*m). (b)* Il-17rc* mRNA was induced after the stimulation of IL-1*β*, IL18, or IL-17A in a dose-dependent manner. (c) IL-17rc protein (green) is weakly expressed in nonstimulated RSCs, but more highly expressed after stimulation with IL-1*β* (100 ng/mL), IL18 (10 ng/mL), or IL-17A (10 ng/mL). The nuclei were stained with DAPI (blue) (scale bar: 20 *μ*m). (d)* Il-17rc* mRNA was induced after the stimulation of 100 ng/mL IL-1*β*, 10 ng/mL IL-18, or 10 ng/mL IL-17A in primary cultured mouse PRE cells. ^*∗*^ *p* < 0.05 compared to control. ^*∗∗∗*^ *p* < 0.001 compared to control.

RSCs cultured in RCM medium maintained spindle-shaped morphology (Figure 1(a)). Because the inflammatory response in RSCs has not yet been characterized, we evaluated expression of Il-17rc, which has been implicated in AMD pathogenesis previously [12, 13]. Indeed,* Il-17rc* mRNA expression was significantly increased in a dose-dependent fashion after stimulation with each cytokine (Figure 1(b)). Further, increased expression of IL-17rc protein was detected after treatment with 100 ng/mL IL-1*β*, 10 ng/mL IL-18, or 10 ng/mL IL-17A, respectively (Figure 1(c)). Interestingly,* Il-17rc* mRNA expression was also significantly increased in primary cultured mouse RPE cells after stimulation with each cytokine (Figure 1(d)).

### 3.2. Proapoptotic Effect of IL-1*β*, IL-18, or IL-17A on RSCs

**Figure 2:**
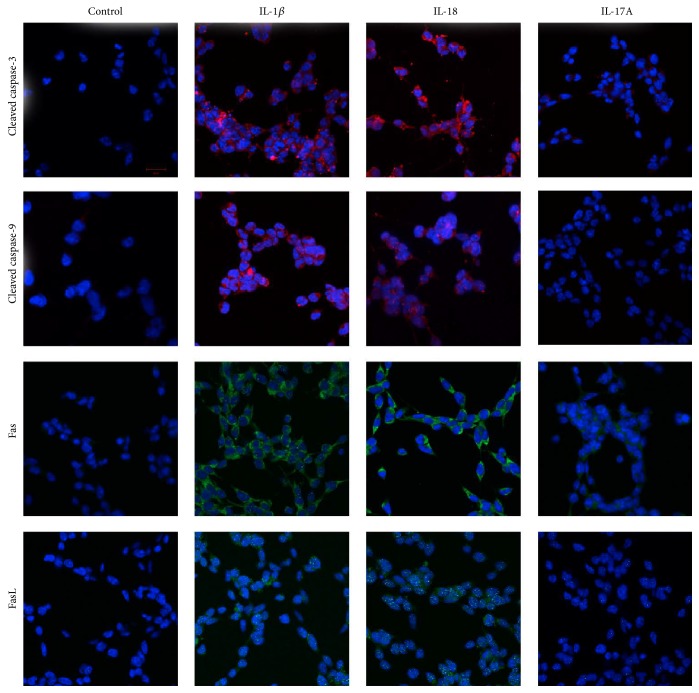
Proapoptotic protein expression in RSCs under stimulation. Immunofluorescence showed higher cleaved caspase-3 (red), cleaved caspase-9 (red), Fas (green), and FasL (green) expression in RSCs under stimulation with IL-1*β* (100 ng/mL) or IL18 (10 ng/mL). IL-17A (10 ng/mL) did not induce these proapoptotic proteins. The nuclei were stained with DAPI (blue) (scale bar: 20 *μ*m).

**Figure 3:**
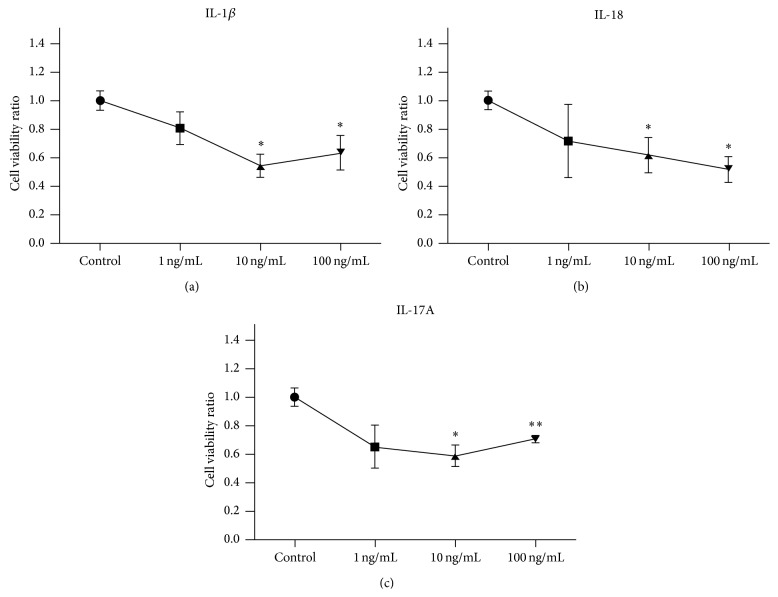
RSCs viability was detected with MTT assay. The RSCs were treated with IL-1*β* (a), IL-18 (b), or IL-17A (c) at different concentrations. ^*∗*^ *p* < 0.05; ^*∗∗*^ *p* < 0.01 compared to control.

In order to test whether IL-1*β*, IL-18, or IL-17A could induce apoptosis in RSCs, cleaved caspase-3, cleaved caspase-9, Fas, and FasL were evaluated by immunohistochemistry. IL-1*β* (100 ng/mL) or IL-18 (10 ng/mL) induced the expression of all the tested proapoptotic proteins when compared to the untreated cells (Figure 2); however, IL-17A had minimal effect on the cells. Accordingly, the MTT assay results demonstrated lower RSC viability in a dose-dependent manner after the cells were treated with IL-1*β* and IL-18. Interestingly, RSCs were also less viable after treatment with IL-17A for 24 hours despite little increase in expression of any proapoptotic proteins (Figure 3).

### 3.3. Ultrastructural Damage in RSCs

**Figure 4:**
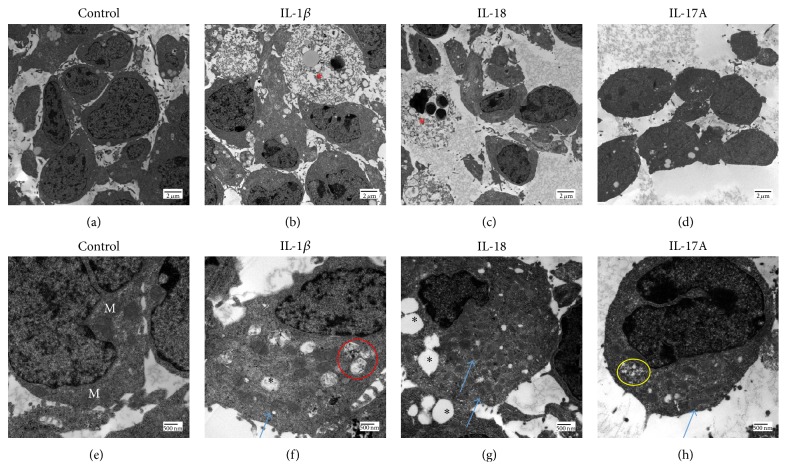
RSC ultrastructure change after stimulation. Control ((a), (e)), 100 ng/mL IL-1*β* ((b), (f)), 10 ng/mL IL-18 ((c), (g)), and 10 ng/mL IL-17A ((d), (h)) (M, mitochondria; black asterisks, cytoplasmic vacuoles; red asterisks, necroptotic cells; blue arrows, degenerated mitochondria; red circle, autophagosome; yellow circle, glycogen deposits).

To further elucidate the subcellular features of RSCs after treatment with IL-1*β*, IL-18, or IL-17A, cellular ultrastructure was examined. With treatment of IL-1*β* (100 ng/mL) or IL-18 (10 ng/mL), the RSCs showed autophagosome formation, mitochondrial degeneration, cytoplasmic vacuoles, and glycogen accumulation (Figure 4). The average number of autophagosomes per cell increased from 1.3 in untreated controls to 9.8, 14.3, and 11 when RSCs were stimulated with IL-1*β*, IL-18, and Il-17, respectively. A few necroptotic and pyroptotic cells with degradation of cytoplasmic contents and chromatin condensations were also noted. IL-17A (10 ng/mL) had a similar effect as IL-18, but to a lesser extent and without necroptosis (Figure 4).

### 3.4. Proinflammatory Effect of IL-1*β*, IL-18, or IL-17A on RSCs

**Figure 5:**
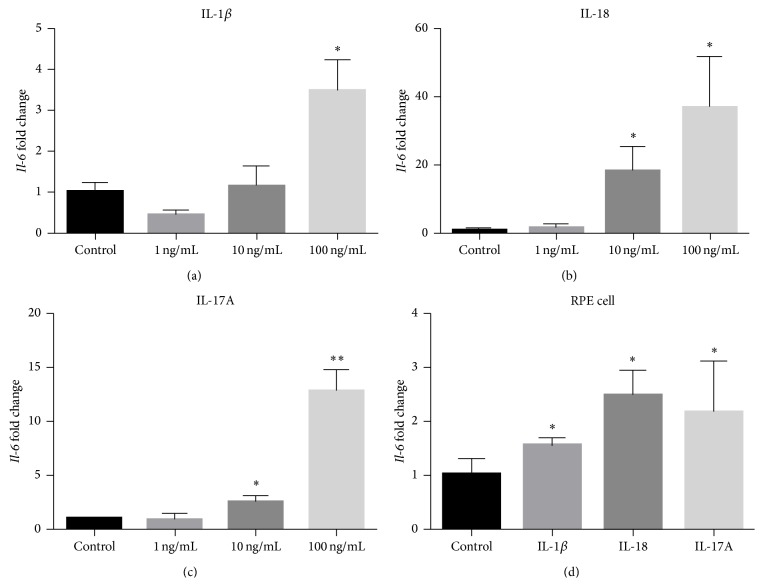
Proinflammatory effect of IL-1*β*, IL-18, or IL-17A.* Il-6* mRNA expression after stimulation with IL-1*β* (a), IL-18 (b), or IL-17A (c) with different concentrations in RSCs. (d)* Il-6* mRNA was induced after the stimulation of 100 ng/mL IL-1*β*, 10 ng/mL IL-18, or 10 ng/mL IL-17A in primary cultured mouse PRE cells. The mRNA levels of the* Il-6* were measured by quantitative RT-PCR. ^*∗*^ *p* < 0.05; ^*∗∗*^ *p* < 0.01 compared to control.

Proinflammatory effects of IL-1*β*, IL-18, and IL-17A were also explored in RSCs. Surprisingly, only the highest concentration of IL-1*β* (100 ng/mL) induced significantly higher expression of* Il-6* transcripts in RSCs (Figure 5(a)). Both IL-18 and IL-17A induced high* Il-6* transcriptsin a dose-dependent manner (Figures 5(b)-5(c)). Consistent with these findings, IL-1*β* (100 ng/mL), IL-18 (10 ng/mL), and IL-17A (10 ng/mL) could induce higher expression of* Il-6* mRNA transcripts in primary cultured mouse RPE cells (Figure 5(d)). However, neither IL-1*β* nor IL-18 could induce detectable* Il-17a* expression from the RSCs (data not shown).

## 4. Discussion

RSCs can be differentiated into many types of retinal cells, including ganglion cells, bipolar cells, and photoreceptor cells. Differentiated photoreceptors from this stem cell line could effectively integrate into* rd1* or* rd7* mouse retinas, improving vision [14]. Recently, the potential for stem cell therapy in AMD has been highlighted [16, 17]. However, no extensive studies on the inflammatory response of RSCs have been performed previously. In our study, we found that RSCs indeed respond to inflammatory stimuli.

Our TEM finding of necroptosis and pyroptosis in the cells stimulated by the cytokines is unique. In contrast to apoptosis, necroptosis requires the function of RIPK3 [18, 19], which regulates the NLRP3 inflammasome [20, 21]. Pyroptosis is a caspase-dependent form of programmed cell death that differs from apoptosis. It depends on the activation of caspase-1 [22]. NLRP3, ASC, and pro-caspase-1 induce caspase-1 activation and can lead to maturation and secretion of IL-1*β* and IL-18. This suggests a link between these two cytokines and pyroptosis/necroptosis, which could be novel pathways for cell death in AMD in addition to apoptosis [23]. Further research on the role of RIPK3 and necroptosis in AMD pathogenesis is warranted.

Our findings of releasing proinflammatory cytokines are in parallel with previous studies [4, 12, 24]. We found that IL-1*β* could induce expression of IL-6 and IL-8 at both the transcript and the protein level in ARPE-19 and human RPE cells, yet this treatment had no effect on cell viability [24]. In our study, IL-1*β* could also induce* Il-6* expression in primary cultured mouse RPE cells and RSCs. However, IL-1*β* upregulated proapoptotic protein expression and decreased cell viability in RSCs, suggesting that IL-1*β* may be more destructive to these cells than to RPE cells. Indeed, the large number of autophagosomes in IL-1*β* treated RSCs supports this conclusion.

Tarallo and colleagues found that intravitreal injection of recombinant IL-18 could induce RPE degeneration in mice, and IL-18 neutralization protected against pAlu-induced RPE degeneration [4]; however, Doyle and her group reported that IL-18 has a protective role in laser induced choroid neovascularization (CNV), as intravitreally injected IL-18-neutralizing antibodies resulted in increased CNV development in mice [5]. These two seemingly conflicting studies may point to diverging roles of IL-18 in RPE versus the myeloid cells and vascular endothelium. Supporting the hypothesis that IL-18 is damaging to the neuroretina, we found that IL-18 decreased cell viability, induced necroptosis/pyroptosis by ultrastructure (Figure 4), and induced proinflammatory response (*Il-6* production) in RSCs. Furthermore, inflammatory response was similarly upregulated in primary cultured RPE cells. Interestingly, it was found that there are increased level of NLRP3 protein,* IL-1β* and* IL-18* mRNA in the RPE of donor eyes from individuals with geographic atrophy and neovascular AMD [4, 25]. Combined with our findings that both IL-1*β* and IL-18 could induce RSCs death* in vitro*, this mechanism may to some extent explain neuroretinal (photoreceptor) atrophy in AMD patients.

IL-17RC serves as an essential subunit of the IL-17 receptor complex and mediates the signal transduction and proinflammatory activities of IL-17A and IL-17F [26], which have been implicated in autoimmune and neurodegenerative diseases [27–29]. Recent research has also implicated the IL-17A/IL-17RC pathway in the pathogenesis of AMD [13, 30]; however, the exact role of IL-17A still remains elusive. In a previous study, we found that IL-17A is cytotoxic to ARPE-19 cells and decreases cell viability. Silencing of IL-17RC could prevent upregulation of cleaved caspase-3 and cleaved caspase-9 and was protective against IL-17A-mediated cell death [12]. In RSCs, IL-17A did not induce measurable proapoptotic proteins but did still decrease cell viability. This may imply that IL-17A-induced effect in RSCs proceeds through pathways other than apoptosis.

One of the most notable roles of IL-17 is its involvement in inducing and mediating proinflammatory response [31]. In synoviocytes, IL-17A could induce IL-6 expression, and knockdown of IL-17RC reversed the effect [32]. Interestingly, we found that not only IL-17A but also IL-1*β* and IL-18 could induce* Il-6 *expression in RSCs and RPE cells. Generally, IL-6 is an important proinflammatory cytokine and has been associated with incidence of early AMD [33]. Furthermore, elevated plasma IL-6 was found in AMD patients with the CC variant in the CFH Y402H polymorphism, indicating a potential role for IL-6 in inflammation-related damage in AMD pathogenesis [34]. It has also been shown that IL-6 can contribute to Th17 cell differentiation from naïve T cells [35]. IL-1*β* combined with IL-23 can promote IL-17 production in naïve and memory T cells [36, 37]. Thus, IL-6 secretion by RSCs or RPE cells could result in a positive feedback loop through which Th17 and *γδ* cells are locally induced. Although neither IL-1*β* nor IL-18 led to increased expression of* Il-17a* in RSCs, both could independently induce* Il-17rc *expression, which may amplify the effect of IL-17A. Interestingly, primary cultured RPE cells could also express notably higher* Il-17rc* and* Il-6 *under the stimulation of IL-1*β*, IL-18, or IL-17A. These findings may account for a potential mechanism of IL-17A-induced pathogenesis in AMD via IL-6 production.

There are some limitations in this study. First, we only explored the response of RSCs, not the differentiated mature neuroretinal cells to IL-1*β*, IL-18, and IL-17A; in future studies, we plan to differentiate the RSCs to photoreceptor cells and explore their response to these cytokines, which will be a better model for photoreceptor changes in AMD. Additionally, this study only evaluates the* in vitro* effects of IL-1*β*, IL-18, and IL-17A; in the future, we hope to explore the effect of these cytokines* in vivo*.

## 5. Conclusions

In conclusion, we demonstrated that IL-1*β*, IL-18, and IL-17A have cytotoxic (necroptosis, pyroptosis, and apoptosis) effect and induce proinflammatory response in RSCs. Inflammasome promotes the maturation of IL-1*β* and IL-18 via caspase-1 activation. Though IL-1*β*, IL-18 alone could not induce IL-17A expression in RSCs, they all induce IL-17RC expression, which may mediate the effect of IL-17A.
